# Effect of statin therapy on mortality from infection and sepsis: a meta-analysis of randomized and observational studies

**DOI:** 10.1186/cc13828

**Published:** 2014-04-11

**Authors:** You-Dong Wan, Tong-Wen Sun, Quan-Cheng Kan, Fang-Xia Guan, Shu-Guang Zhang

**Affiliations:** 1 Department of Integrated ICU, the First Affiliated Hospital of Zhengzhou University, 1 Jianshe East Road, Zhengzhou 450052, China; 2 Pharmaceutical Department, the First Affiliated Hospital of Zhengzhou University, Zhengzhou, PR China; 3 Academy of Medical Science, Henan Province, Zhengzhou, PR China

## Abstract

**Introduction:**

Observational data have suggested that statin therapy may reduce mortality in patients with infection and sepsis; however, results from randomized studies are contradictory and do not support the use of statins in this context. Here, we performed a meta-analysis to investigate the effects of statin therapy on mortality from infection and sepsis.

**Methods:**

We searched electronic databases (PubMed and Embase) for articles published before November 2013. Randomized or observational studies reporting the effects of statin therapy on mortality in patients with infection or sepsis were eligible. Randomized and observational studies were separately pooled with relative risks (RRs) and random-effects models.

**Results:**

We examined 5 randomized controlled trials with 867 patients and 27 observational studies with 337,648 patients. Among the randomized controlled trials, statins did not significantly decrease in-hospital mortality (RR, 0.98; 95% confidence interval (CI), 0.73 to 1.33) or 28-day mortality (RR, 0.93; 95% CI, 0.46 to 1.89). However, observational studies indicated that statins were associated with a significant decrease in mortality with adjusted data (RR, 0.65; 95% CI, 0.57 to 0.75) or unadjusted data (RR, 0.74; 95% CI, 0.59 to 0.94).

**Conclusions:**

Limited evidence suggests that statins may not be associated with a significant reduction in mortality from infection and sepsis. Although meta-analysis from observational studies showed that the use of statins was associated with a survival advantage, these outcomes were limited by high heterogeneity and possible bias in the data. Therefore, we should be cautious about the use of statins in infection and sepsis.

## Introduction

Sepsis is a complex syndrome caused by an uncontrolled systemic inflammatory response to infection. The manifestations of sepsis are multifaceted and ultimately result in multi-organ dysfunction [1]. When accompanied by evidence of hypoperfusion or dysfunction of at least one organ system, sepsis progresses to “severe sepsis”. Moreover, if accompanied by hypotension or a need for vasopressors, the conditions further worsen to “septic shock” [2]. Increasing severity correlates with increasing mortality, which increases from 25% to 30% for severe sepsis up to 40% to 70% for septic shock, and even if patients survive the acute phase of sepsis, clinical data indicate that surviving patients have higher mortality rates than patients who have not had sepsis [2-5].

Statins are used to lower cholesterol levels, and their cardiovascular benefits are widely accepted in medical practice. In addition, statins also have a wide variety of properties that are independent of their lipid-lowering ability, termed “pleiotropic effects” [6-8]. Many observational studies have demonstrated a significant protective effect from statins in patients with sepsis, and previous meta-analysis showed a similar outcome [9,10]. However, at the time of that analysis, no appropriate studies describing the therapeutic effects of statins in randomized controlled trials had been published; therefore, we do not know whether statins truly have beneficial effects, or whether these results were observed due to the bias effect. One meta-analysis [11] evaluating the prophylactic effects of statins found that statins did not reduce the risk of infections. However, this meta-analysis included only randomized controlled trials (RCTs) and did not describe the therapeutic effects of statins.

Because of the limited quantity and possible heterogeneity of RCTs, we also searched relevant observational studies as a supplement. Thus, in this manuscript, we present a meta-analysis of randomized and observational studies to investigate the effects of statin therapy on mortality from infection and sepsis.

## Materials and methods

The systematic review and meta-analysis were performed according to the recently published Preferred Reporting Items for Systematic Reviews and Meta-Analyses (PRISMA) statement [12]. Ethical approval and patient consent are not required since this is a meta-analysis of previously published studies.

### Literature search and inclusion criteria

Electronic databases, including PubMed and Embase were searched from inception to November 2013 to identify relevant studies. We used a combination of keywords related to the type of statin (“hydroxymethylglutaryl coenzyme A reductase inhibitors” or “anticholesteremic agents” or “statin” or “simvastatin” or “rosuvastatin” or “pravastatin” or “atorvastatin” or “fluvastatin” or “cerivastatin” or “pitavastatin” or “lovastatin”) and the type of infection-associated disease (“infection” or “sepsis” or “bacteremia” or “pneumonia”). An English language restriction was imposed. We also evaluated the references in relevant review articles and meta-analyses to identify other potentially eligible studies. An RCT or an observational study was included if it met the following criteria: adult patients experienced infection or sepsis, statins compared with a control and data available on the mortality.

### Data extraction and quality assessment

Data were extracted independently by two investigators (YDW and TWS). Discrepancies were resolved by consensus or a third author’s (FXG) adjudication. We separately extracted and pooled data from RCTs and observational studies. For RCTs, the following data were abstracted from each study: characteristics of the studies, characteristics of the included patients and outcomes of the studies. For observational studies only, we also extracted types of effect sizes (odds ratio (OR) or hazard ratio (HR)), adjusted covariates, data sources, financial support, study period and conclusion of the trials (see Additional files 1, 2 and 3). For RCTs, the primary endpoint was mortality (in-hospital mortality and 28-day mortality). The secondary endpoints were the rates of mechanical ventilation (MV) usage, ICU admission and newly developed severe sepsis. For observational studies, we pooled the mortality (in-hospital mortality, 15-day mortality, 30-day mortality and 90-day mortality) with adjusted and unadjusted data.

The methodological quality of RCTs was evaluated using the Jadad scale [13,14]. Because the observational studies were only used as a supplement for the RCTs, we did not evaluate the quality of the observational studies.

### Data analysis

In examining the associations between statins and infection/sepsis mortality, results were expressed as relative risks (RRs) with 95% confidence intervals (CIs); risk ratios (RRs), ORs and HRs were included as eligible RRs without distinction because each provided effect sizes of similar magnitude [15]. Heterogeneity across trials was assessed using a standard chi-squared test, with significance being set at *P* <0.10. Heterogeneity was also assessed by means of the I^2^ statistic, with significance being set at I^2^ > 50%. The random effects model was used for statistical analysis due to wide clinical and methodological variability across the trials. We further conducted subgroup analyses to explore possible explanations for heterogeneity. Publication biases were evaluated using Funnel plots and Egger’s tests [16]. Statistical analysis was performed using STATA 12.0 (Stata Corp, Tong-Wen Sun, Zhengzhou, Henan province, China). A *P-*value of less than 0.05 was considered to represent statistical significance.

## Results

### Study identification and selection

**Figure 1:**
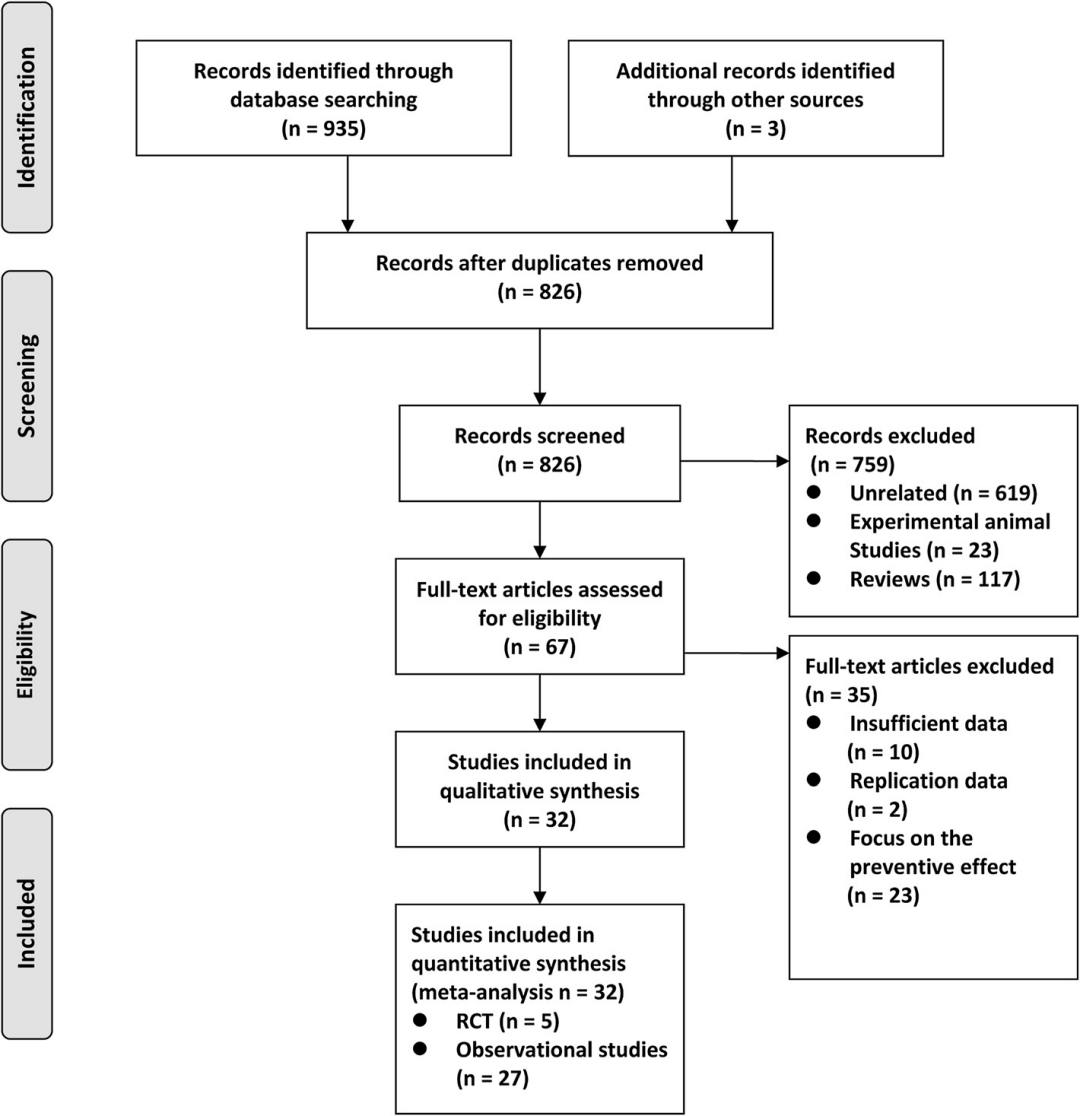
Flow diagram for selection of articles.

A total of 826 titles and abstracts were identified after removal of duplicate studies in the primary search; 619 were excluded as unrelated, 23 were excluded as experimental animal studies, and an additional 117 were excluded as reviews. A total of 67 articles were fully read, and of these, 35 were excluded for other reasons listed in the flow diagram. Overall, 5 RCTs [17-21] and 27 observational studies [22-48] met the eligibility criteria and were included (see the detail in Figure 1). We excluded the Justification for the Use of Statins in Prevention: an Intervention Trial Evaluating Rosuvastatin (JUPITER) trial [49] and the study by Makris *et al.*[50] because these trials only included individuals without any signs of infection or sepsis and focused on the preventive effects of statins instead of their therapeutic effects.

### Study characteristics

The study characteristics and Jadad scores of the five RCTs [17-21] included for review appear in Tables 1, 2 and 3 and the characteristics, outcomes, adjusted covariates and so on of the 27 observational studies [22-48] are summarized in Additional files 1, 2 and 3. In total, 338,515 patients (867 RCT patients and 337,648 observational patients) were included. Of the RCTs, all were published between 2009 and 2013. The sample sizes of the RCTs ranged from 83 to 284. The populations of two trials [20,21] were from ICUs, and those of the remaining RCTs [17-19] were from general wards; thus, there was a large amount of variation in the severity of the illness, from mean Acute Physiology and Chronic Health Evaluation II (APACHE II) scores of 8.9 to 23.5. Among the five RCTs included here were all reported mortality events (in-hospital mortality [17,18,20,21] and 28-day mortality [19-21]) and length of hospital stay events [17-21]. Regarding the types and doses of statins, two studies administered atorvastatin 20 mg daily [18,20], one study administered atorvastatin 40 mg daily [19], and the remaining two studies administered simvastatin 20 mg daily [17] or 60 mg daily [21]; all control groups were given a placebo. The quality of the included studies was assessed by Jadad scores, and the median Jadad score of the included studies was 4 (the range was 4 to 5).

**Table 1:** Main characteristics of RCTs included in the meta-analysis of statins for infection and sepsis

**Study/year**	**Country**	**Study design**	**No. total (statins/non-statins)**	**Patient characteristics**	**Jadad score**	**Statins group**	**Control group**
Novak V *et al.*[17]/2009	Israel	Double-blind placebo controlled randomized clinical trial.	83 (42/41)	Not receiving statin therapy during the three months preceding the index admission, within 12 h of admission to a general medical ward with a suspected or documented bacterial infection, and prescribed intravenous antibiotics therapy by their physicians.	5 points	40 mg of simvastatin orally immediately following enrollment, and daily 20 mg of simvastatin after that, until hospital discharge or the development of severe sepsis.	Placebo
Kruger PS *et al.*[18]/2011	Australia	Prospective randomized double-blind placebo-controlled trial	150 (75/75)	Patients with any of 97 potential infection-related diagnoses; admitted to both the general wards or the intensive care unit; preexisting statin therapy and the treating physician prepared to either continue or discontinue this therapy	5 points	20 mg of atorvastatin orally or via nasogastric tube at the earliest opportunity and was continued daily for the duration of hospital admission up to a maximum of 28 days.	Placebo
Patel JM *et al.*[19]/2012	UK	phase II randomized double-blind placebo-controlled trial	100 (49/51)	Documented new proven or suspected infection and the presence of any two of the signs and symptoms of infection (white blood cell >11 or <4 × 109/L, temperature >38°C or <36°C, heart rate >90 bpm, or respiratory rate >20/minute) for less than 24 hours.	4 points	Atorvastatin 40 mg daily was administered orally within 24 hours of randomization. Treatment continued for the duration of their hospital stay or 28 days if earlier	Placebo
Kruger P *et al.*[20]/2013	Australia	Phase II, prospective, randomized, double-blind, placebo controlled trial	250 (123/127)	Critically ill, had strongly suspected or proven infection, fulfilled three or more of the features of systemic inflammatory response syndrome within the 48 hours and had an organ dysfunction of less than 24 hours duration	4 points	Take statins more than two weeks prior to hospital admission; Atorvastatin 20 mg was orally or via nasogastric tube given following randomisation then continued daily until Day 14 or until death or discharge from the intensive care unit	Placebo
Papazian L *et al.*[21]/2013	France	Randomized, placebo-controlled, double-blind parallel-group trial	284 (146/138)	Received mechanical ventilation in the intensive care unit for at least two days and had suspected ventilator-associated pneumonia, statins therapy started on the same day as antibiotic therapy	5 points	Simvastatin 60 mg given via a nasogastric tube or orally from study inclusion to ICU discharge, death or Day 28	Placebo

**Table 2:** Outcome data of RCTs included in the meta-analysis of statin for infection and sepsis

**Study/year**	**Mean age (y) statin/ non-statin (mean ± SD)**	**Male sex (%) statin/non-statin**	**Need for MV or ICU admission**	**Severity of illness statin/non-statin (mean ± SD)**	**Mortality statin/non-statin**		**Length of hospital stay statin/non-statin**	**New development of severe sepsis**
					In-hospital mortality	28-day mortality		
Novak V *et al.*[17]/2009	66.0 ± 17.2/72.7 ± 13.8	35.7/39.0	3/42 vs 5/41	APACHE II score (11.2 ± 4.3/8.9 ± 4.8)	0/42 vs 0/41	NA	4 days (IQR 3 to 6)/4 days (IQR 2 to 6)	2/42 vs 2/41
Kruger PS *et al.*[18]/2011	68.2 ± 12.7/68.5 ± 11.9	64.0/65.0	13/75 vs 11/75	NA	6/75 vs 4/75	NA	7 days (IQR 4 to 12)/6 days (IQR 4 to 11)	NA
Patel JM *et al.*[19]/2012	62.8 ± 21.2/64.0 ± 15.6	51.0/51.0	0/49 vs 2/51	APACHE II score (11.8 ± 5.6/11.9 ± 6.0)	NA	2/49 vs 2/51	5 days (IQR 3 to 13)/6 days (IQR 4 to 12)	2/49 vs 12/51
Kruger P *et al.*[20]/2013	58 (44 to 67)/64 (50 to 75)*	59.0 /65.0	123/123 vs 127/127	APACHE II score (22.1 ± 7.7/23.5 ± 7.8)	16/123 vs 23/127	12/123 vs 22/127	18.3 days (IQR 11.0 to 38.7)/22.7 days (IQR 12.4 to 37.0)	NA
Papazian L *et al.*[21]/2013	60.0 ± 16.0/59.0 ± 17.0	73.0/77.0	146/146 vs 138/138	SOFA score (7.2 ± 3.6/6.7 ± 2.9)	43/146 vs 38/138	31/146 vs 21/138	37 days (IQR 21 to 59)/35 days (IQR 22 to 62)	NA

*Data were shown as median (IQR). APACHE II, Acute Physiology and Chronic Health Evaluation II; ICU, intensive care unit; IQR, interquartile range; SOFA, sepsis-related organ failure assessment.

**Table 3:** Methodological quality assessment (risk of bias) of included studies by Jadad scale

**Study/year**	**Randomization (0 to 2 point(s))**			**Blinding (0 to 2 point(s))**			**Withdrawals and dropouts (0 to 1 point)**		**Total score (0 to 5 point(s))**
	**No randomization or inappropriate randomization (0 point)**	**Described as randomized (1 point)**	**Method of randomization was described and appropriate (2 point)**	**No blind or inappropriate method of blinding (0 point)**	**Described as double blind (1 point)**	**Method of double blinding was described and appropriate (2 point)**	**Did not describe the follow-up (0 point)**	**A description of withdrawals and dropouts (1 point)**	
Novak V *et al.*[17]/2009	-	-	**	-	-	**	-	*	5
Kruger PS *et al.*[18]/2011	-	-	**	-	-	**	-	*	5
Patel JM *et al.*[19]/2012	-	-	**	-	*	-	-	*	4
Kruger P *et al.*[20]/2013	-	-	**	-	*	-	-	*	4
Papazian L *et al.*[21]/2013	-	-	**	-	-	**	-	*	5

One“*”stands for one point, “**” indicated two point.

### Result from RCTs

**Figure 2:**
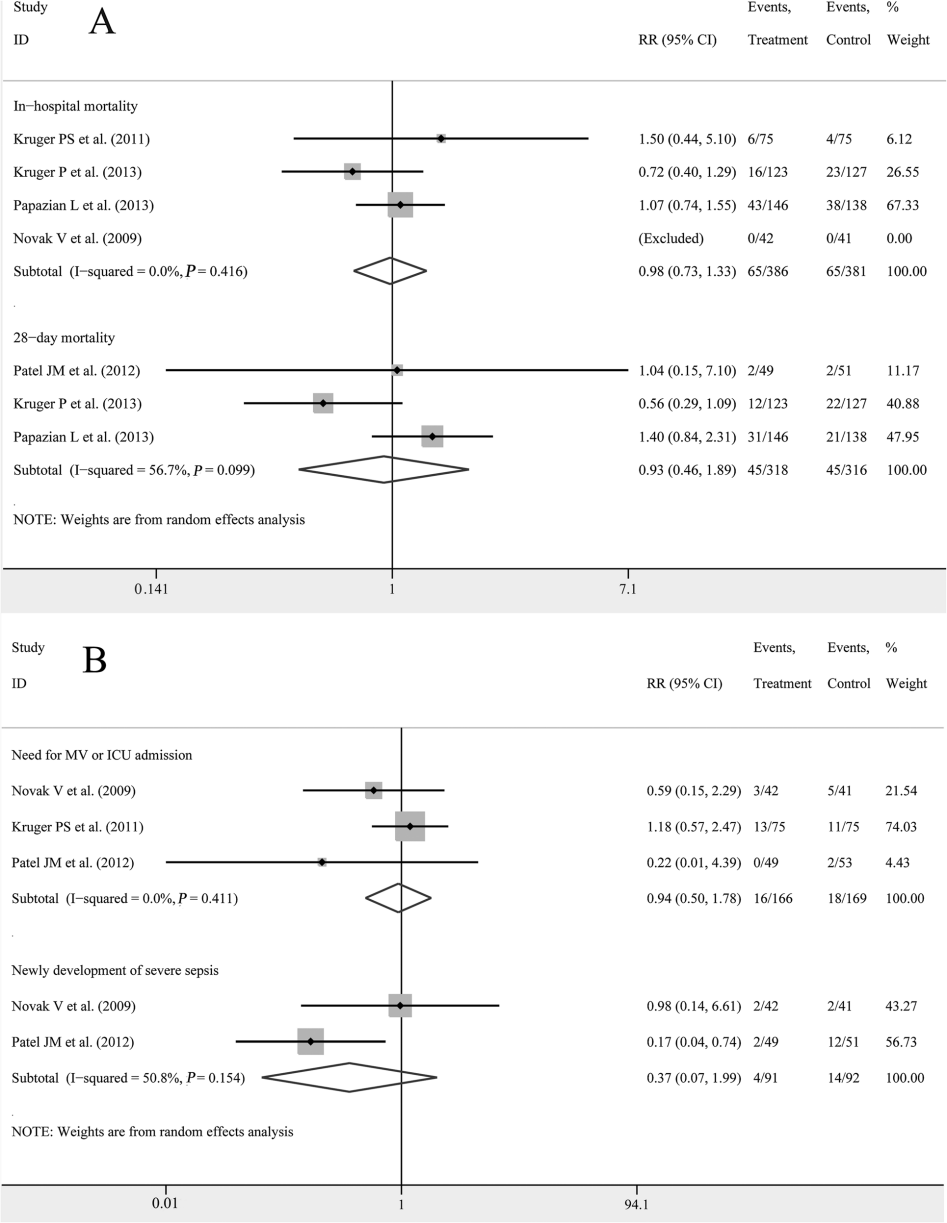
**Forest plot of randomized controlled trials. A**. This is a forest plot for the relative risk of in-hospital mortality and 28-day mortality from randomized controlled trials. **B**. This is a forest plot for the rate need for MV or ICU admission and the rate of new development of severe sepsis from randomized controlled trials.

Figure 2A shows the pooled results from the random-effects model combining the RRs for mortality. Overall analysis from RCTs with 767 patients total from four studies [17,18,20,21] showed that there was no significant difference between statins (65/386) and placebo (65/381) in terms of in-hospital mortality (RR, 0.98; 95% CI, 0.73 to 1.33), with low heterogeneity among the studies (*I*^*2*^ = 0.0%, *P* = 0.42). Similarly, using three studies [19-21] with 634 patients, no 28-day mortality advantage was observed in the group receiving statins (statins 45/318, placebo 45/316; RR, 0.93; 95% CI, 0.46 to 1.89), with moderate heterogeneity among the studies (*I*^*2*^ = 56.7%, *P* = 0.10)*.*

Based on a meta-analysis of three studies [17-19] with 335 participants, there was no difference in the rates of mechanical ventilation (MV) usage or ICU admission for patients receiving statins (16/166) and placebo (18/169; RR, 0.94; 95% CI, 0.50 to 1.78), with low heterogeneity between studies (*I*^*2*^ = 0.0%, *P* = 0.41). We also performed a meta-analysis of the rate of newly developed severe sepsis [17,19] and found that compared with placebo (14/92), statins (4/91) did not have a beneficial effect (RR, 0.37; 95% CI, 0.07 to 1.99); significant heterogeneity was found among these two studies (*I*^*2*^ = 50.8%; *P* = 0.15; Figure 2B).

### Results from observational studies

**Figure 3:**
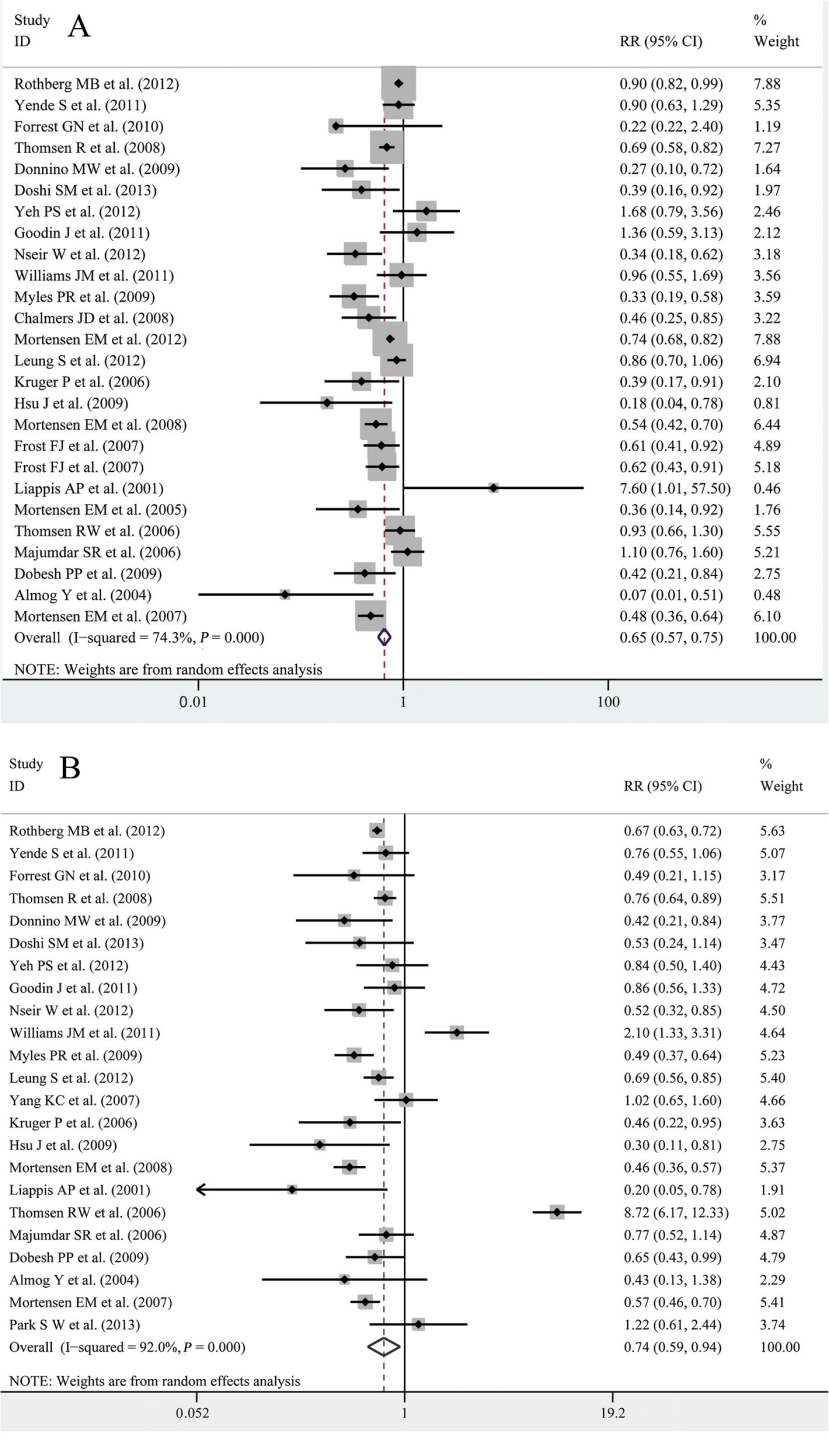
**Forest plot of observational cohort studies A.** A forest plot for the relative risk of mortality from observational cohort studies with adjusted data. **B**. A forest plot for the relative risk of mortality from observational cohort studies with unadjusted data.

In a meta-analysis with 337,648 patients and 26 studies included, statins were associated with a significant decrease in mortality with adjusted data (RR, 0.65; 95% CI, 0.57 to 0.75) and unadjusted data (RR, 0.74; 95% CI, 0.59 to 0.94); high heterogeneity was found with adjusted data (*I*^*2*^ = 74.3%; *P* <0.01; Figure 3A) and unadjusted data (*I*^*2*^ = 92.0%; *P* <0.01; Figure 3B). The outcome was different from that of our meta-analysis of RCTs. In order to explore the reasons for these differences, we carried out a subgroup analysis according to the type of effect size (HR or OR), type of mortality, statin exposure (current or former statin user), financial support and propensity score. When comparing patients using statins to those not using statins, all subgroups except one (90-day mortality: RR, 0.93; 95% CI, 0.73 to 1.19) supported that statins provided a survival advantage in infection and sepsis. The detailed results are shown in Table 4.

**Table 4:** Subgroup analyses of observational studies

**Subgroup**	**No. studies**	**Total sample size**	**RR (95% CI)**	**Heterogeneity *I***^***2***^
Total [22-35,37-47]	26	336,245	0.65 (0.57 to 0.75)	74.3%
**Type of effect size**				
OR [22-26,29,31,33,34,37-47]	20	253,013	0.67 (0.57 to 0.78)	74.0%
HR [27,28,30,32,35,40]	6	83,232	0.59 (0.38 to 0.91)	78.6%
**Type of mortality**				
In-hospital mortality [22,26,29,37,40,41,44,45]	9	259,052	0.72 (0.54 to 0.96)	70.1%
90-day mortality [23,28,35]	3	4,548	0.93 (0.73 to 1.19)	29.2%
30-day mortality [24,25,27,30-34,39,42,43,47]	12	71,973	0.57 (0.48 to 0.69)	69.6%
28-day mortality [46]	1	361	0.07 (0.01 to 0.51)	-
15-day mortality [38]	1	311	0.18 (0.04 to 0.78)	-
**Statin exposure**				
Current statin user [22,24,26-29,32,35,38,40,41], [45]	12	186,004	0.64 (0.48 to 0.85)	75.7%
Former statin user [23,25,30,31,33,34,37,39], [40,42-44,46,47]	14	150,241	0.65 (0.55 to 0.77)	67.5%
**Sample size**				
<1,000 [24,27-30,37,38,41,42,45,46]	11	4,266	0.49 (0.29 to 0.83)	69.5%
≥1,000 [22,23,25,26,31-35,39,40,43], [44,47]	15	331,979	0.70 (0.61 to 0.80)	75.1%
**Financial support**				
YES [22,23,25,28,29,31,32,34], [35,38,39,44,47]	13	194,211	0.75 (0.64 to 0.87)	78.1%
NO [24,27,30,37,42,43]	6	7,289	0.43 (0.26 to 0.73)	67.6%
NA [26,33,40,41,45,46]	7	134,745	0.51 (0.33 to 0.76)	57.9%
**Data sources**				
Database [22,25,32,34,39,40,43,47]	9	318,845	0.66 (0.56 to 0.77)	81.3%
Registry [23,24,26-31,33,35,37,38,41], [42,44-46]	17	17,400	0.61 (0.45 to 0.81)	70.6%
**Adjusted by propensity score**				
YES [22,23,25,28,31,34,35,39], [42,44]	10	187,420	0.80 (0.70 to 0.91)	71.1%
NO [24,26,27,29,30,32,33,36-38], [40,41,43,45-47]	16	148,825	0.49 (0.38 to 0.64)	62.5%

Pooled with adjusted outcomes of included studies and random effects model. Frost *et al*. [40] including a matched cohort study and a separate case-control study had been counted as two studies. HR = Hazard Ratio; OR = Odds Ratio; RR, Relative Risk; 95% CI = 95% Confidence Intervals.

### Publication bias

**Figure 4:**
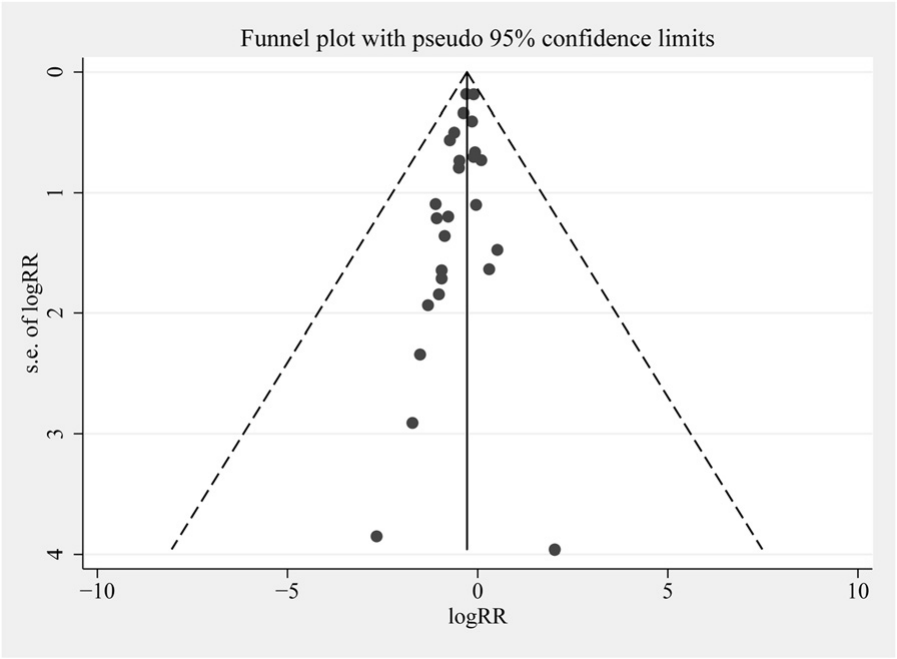
**Funnel plot for the risk of mortality from observational cohort studies with adjusted data.** RR, relative risk; s.e., standard error.

The quantity of RCTs was not sufficient to evaluate publication bias, so we only assessed the results of observational studies. Egger’s test (*P* = 0.025) suggested that we may have encountered publication bias. Also, there was asymmetry in the middle and lower segments of the funnel plot in which small negative trials were missing (Figure 4), potentially leading to overstatement of the treatment effect.

## Discussion

In this meta-analysis, the beneficial effects of statin treatment on mortality resulting from infection and sepsis were not found from RCTs and in one of the subgroups from the set of observational studies; these data were quite different from previously reported meta-analyses [9,10]. However, while our meta-analysis of observational studies yielded a contradictory outcome, this may have resulted from the high heterogeneity and possible publication bias associated with these observational studies. This may indicate that the “pleiotropic effects” of statins may not be suitable for treatment of infection and sepsis.

The main findings from our meta-analysis of RCTs were inconsistent with those of observational studies. A major reason for this inconsistency may be the methodological differences between RCTs and observational studies. Observational studies are always at risk of unmeasured confounding variables, which are inherent to the study design and cannot be avoided. Among these biases, “healthy user” effects are a major source of such potentially confounding variables. In a population-based prospective cohort study by Majumdar *et al*. [44], this assertion was supported by a propensity score. They observed that statin users were more likely to be former smokers and have up-to-date immunizations for pneumococcus and influenza and less likely to need advanced directives. Another possible explanation is that most observational studies primarily focus on statins as prophylaxis whereas the RCTs seem to evaluate statins as therapy. In observational studies, most patients receive statin therapy before their inpatient hospital course whereas RCT patients receive statin therapy after the onset of sepsis. Except for “healthy user” effects, other biases also exist in observational studies, including socioeconomic and health statuses and so on [51]. Such biases are not present in meta-analyses of data from only RCTs. Based on this perspective, our meta-analysis of RCTs only demonstrated that statins have no therapeutic effects on infection and sepsis, and this is probably the more realistic result. Another limitation of meta-analyses of observational studies was the significant heterogeneity. We performed a subgroup analysis; however, we were still unable to completely remove heterogeneity. This may indicate that the most likely cause of heterogeneity was the type of statin therapy (different doses and types of statins) and the severity of the various infections (the APACHE II scores varied considerably across trials), which could not be adjusted for because of an insufficient amount of data. In spite of the disadvantage of observational studies, observational studies do provide valuable information. Observational studies are crucial to understanding how clinical trial-defined therapies are incorporated into routine clinical practice. In this manner, effectiveness as well as potentially uncommon adverse effects of statins can be examined over extended periods of time in “real-world” populations [52].

To the best of our knowledge, this is the first meta-analysis to explore the effects of statin therapy on mortality from infection and sepsis with high-quality RCT and observational data. Most previous meta-analyses have focused on observational studies [9,10] because of the limitations of slow publication associated with RCTs. A meta-analysis conducted in 2010 by Janda *et al*. [10], which included 19 observational studies and 1 RCT [53], found that statins provided a protective effect; however, we did not include this RCT [53] because they only included patients after aneurysmal subarachnoid hemorrhage and infections were related to surgical complications. Additionally, the population did not meet our standards, and the researchers investigated preventive effects of statins instead of treatment effects. Additionally, in the meta-analysis by Janda *et al*. [10], they found an asymmetrical funnel plot, but did not observe significant publication bias as assessed with Egger’s test (*P* = 0.052). In contrast, our analysis included eight more studies, and the publication bias was significant, exhibiting both an asymmetrical funnel plot and a significant Egger’s test result (*P* = 0.025). Uncorrectable heterogeneity, established publication bias, residual confounding variables, and healthy-user bias have always been deep concerns of investigators. These issues were examined more thoroughly in our meta-analysis, which had the greatest number of observational studies. Fortunately, our meta-analysis of RCTs removed most of these problems, and our results may provide some insights into further studies. More recently, a meta-analysis [54] including five RCTs found statin therapy has no effect on mortality in septic patients. In order to control the overall quality, we excluded one conference paper included in this meta-analysis, and included one more large RCT [21] and 27 observational studies [22-48]; thus, we found an interesting contradictory outcome between RCTs and observational studies.

Our meta-analysis of observational studies verified several previously proposed hypotheses suggested by RCTs. According to a study by Kruger *et al*. [20], for the cohort as a whole there was no statistically significant difference in mortality. However, atorvastatin therapy in prior statin users was associated with lower baseline interleukin (IL)-6 levels and a statistically significant improvement in 28-day mortality, but this survival advantage did not appear in patients with no prior statin use. Similar outcomes were observed by Shyamsundar *et al*. [55]. Therefore, Kruger *et al*. [20] hypothesized that prior continued statin therapy may be associated with a significant special biological effect in critically ill patients with infection. It was further hypothesized that some of the pathways associated with the inflammatory response caused by infection and sepsis could be modulated by prior statin therapy [20]. In order to test this theory, we conducted a special subgroup analysis with current statin users and former statin users. We observed that no clear differences were found between these groups, and current statin users still exhibited a survival advantage. We also conducted a novel subgroup analysis related to financial support, which was not investigated in previous meta-analyses. We expected that sources of financial support may be an important bias and may explain our observed publication bias. Unfortunately, the results were not consistent with our initial hypothesis, and there was no clear difference between studies with and without financial support.

### Limitations of the study

One important limitation of this meta-analysis was the low number and sample size of RCTs, which made it difficult to carry out a subgroup analysis, and, thus, it was under-powered to detect a completely reliable conclusion. One important reason for the small sample size may have been the high refusal rate [17] and slow recruitment [17-19] associated with RCTs. Another limitation to our meta-analysis was that we were unable to assess the impact of statins on other clinically meaningful end points because of the unsuitable pooled format of the data, such as length of hospital stay.

## Conclusions

According to our analysis, the limited evidence suggested that statins may not be associated with a significant reduction in mortality from infection and sepsis. Though meta-analysis from observational studies showed a survival advantage of statins, these results were limited by the high heterogeneity and possible bias of the observational studies used in the analysis. Therefore, we should be cautious about the use of statins in patients with infection and sepsis.

## Key messages

• Limited evidence from meta-analysis of RCTs suggested that statins may not be associated with a significant reduction in mortality from infection and sepsis.

• Meta-analysis of observational studies showed the use of statins was associated with a survival advantage. However, these outcomes were limited by high heterogeneity and possible bias.

• We should be cautious about the use of statins in patients with infection and sepsis.

## Abbreviations

95% CI: 95% Confidence intervals; APACHE II: Acute Physiology and Chronic Health Evaluation II; HR: Hazard ratio; MV: Mechanical ventilation; OR: Odds ratio; RCT: Randomized controlled trial; RR: relative risk.

## Competing interests

The authors declare that they have no competing interests.

## Authors’ contributions

YDW conceived and designed the study, did the literature search and the acquisition of data, analyzed and interpreted data, and drafted and critically revised the manuscript for important intellectual content. YDW and TWS did the literature search and the acquisition of data, and drafted and critically revised the manuscript for important intellectual content. QCK conceived and designed the study, drafted and critically revised the manuscript for important intellectual content, supervised the study, and gave administrative, technical or material support. FXG and SGZ conceived and designed the study, statistically analyzed and interpreted the data, drafted and critically revised the manuscript for important intellectual content, supervised the study, and gave administrative, technical or material support. All authors read and approved the final version of the manuscript.

## Supplementary Material

Additional file 1: Table S1 Characteristics of observational studies included in the meta-analysis. Click here for file

Additional file 2: Table S2 Outcomes of observational studies reviewed. Click here for file

Additional file 3: Table S3 Main outcomes of observational studies reviewed. Click here for file
