# Supplementary material for: Effect of statin therapy on mortality from infection and sepsis: a meta-analysis of randomized and observational studies
Source: Crit Care. 2014 Apr 11;18(2):R71. doi: 10.1186/cc13828 (PMC4056771; doi:10.1186/cc13828)
Supplement: Additional file 1: Table S1 — Characteristics of observational studies included in the meta-analysis. [file cc13828-S1.doc]

**Additional table 1. Characteristics of Observational Studies Included in the Meta–analysis**

| **Study/Year** | **Country** | **Data sources** | **Financial Support** | **Design** | **Patients [n]** | | | **Study Period** |
| --- | --- | --- | --- | --- | --- | --- | --- | --- |
|  |  |  |  |  | **Total** | **Statins** | **Nonstatins** |  |
| Rothberg MB et al [22]/2012 | USA | Premier’ s Perspective Database | YES | Observational cohort study | 121,254 | 23,285 | 97,969 | January 2003 – December 2005 |
| Yende S et al[23]/ 2011 | USA | Registry of emergency departments of 28 US hospitals | YES | Multicenter inception cohort study | 1895 | 426 | 1469 | December 2001 – November 2003 |
| Forrest GN et al[24]/2010 | USA | Registry of a medical center | NO | Retrospective matched– cohort study | 45 | 15 | 30 | January 2003 –  December 2006 |
| Thomsen RW et al[25]/2008 | Denmark | Health databases | YES | Population–Based Cohort Study | 29,900 | 1,372 | 28,528 | January 1997–  December 2004 |
| Donnino MW et al[26]/2009 | USA | A secondary analysis of a cohort study | NA | Prospective, observational cohort study | 2,036 | 474 | 1,562 | December 2003–  September 2004 |
| Doshi SM et al[27] 2013 | USA | Registry of a medical center | NO | Retrospective cohort study | 347 | 90 | 257 | January 2000–  June 2010 |
| Yeh PS et al[28]/2012 | Taiwan | Registry of a hospital | YES | Prospective observational cohort study | 514 | 121 | 393 | August 2006 –  July 2010 |
| Goodin J et al[29]  /2011 | USA | Registry of two medical center | YES | Retrospective cohort study | 568 | 124 | 444 | 2001 –2007 |
| Nseir W et al[30] /2012 | Israel | Registry of one medical center | NO | Retrospective study | 319 | 158 | 161 | January 2003 –  December 2009 |
| Williams JM et al[31]/2011 | Australia | Registry of one hospital | YES | Prospective observational study | 2,642 | 395 | 2,247 | October 2007 –November 2008 |
| Myles PR et al [32]/2009 | UK | The health  improvement network database | YES | Population–based cohort study | 3,681 | 357 | 3,324 | July 2001 –  July 2002 |
| Chalmers JD et al[33]/2008 | UK | Registry of one hospital | NA | Prospective observational study | 1,007 | 549 | 458 | January 2005–  November 2007 |
| Mortensen EM et al[34] /2012 | USA | Veterans Affairs Health Care System administrative databases. | YES | Retrospective cohort study | 16,222 | 4,724 | 11,498 | October 2001–  September 2007 |
| Leung S et al[35]/2012 | USA | Registry of two tertiary hospitals | YES | Retrospective cohort study | 2,139 | 592 | 1,547 | January 2008 –  December 2009 |
| Yang KC et al[36] /2007 | Taiwan | Registry of one hospital | NA | Retrospective study | 454 | 104 | 350 | January 2001–  December 2002 |
| Kruger P et al[37] 2006 | Australia | Registry of one hospital | NO | Retrospective cohort analysis | 438 | 66 | 372 | January 2000 –  December 2003 |
| Hsu J et al[38]/2009 | USA | Registry of one hospital | YES | Retrospective cohort study | 311 | 78 | 233 | January 1995 – November 2006 |
| Mortensen EM et al[39]/2008 | USA | Veterans Affairs Health Care System administrative | YES | Retrospective national cohort study | 8,652 | 1,567 | 7,085 | July 1999 –  January 2000 |
| Frost FJ et al[40]/ 2007 | USA | The Lovelace Patient Database | NA | Matched cohort study | 76,232 | 19,058 | 57,174 | January 1992 –December 2003 |
| Frost FJ et al[40]/2007 | USA | The Lovelace Patient Database | NA | Case–control studies | 54,533 | 397 | 54,136 | January 1992 –December 2003 |
| Liappis AP et al[41]/ 2001 | USA | Registry of one hospital | NA | Retrospective review | 388 | 35 | 353 | September 1995  –April 2000. |
| Mortensen EM et al[42] /2005 | USA | Registry of two teaching hospitals. | NO | Retrospective cohort study | 787 | 110 | 677 | January 1999 –December 2002 |
| Thomsen RW et al[43]/2006 | Denmark | National Database | NO | Prospectively cohort study | 5,353 | 176 | 5,177 | January 1997–  December 2002 |
| Majumdar SR et al[44]/2006 | Canada | Registry of six hospitals | YES | Population based prospective cohort study. | 3,415 | 325 | 3,090 | 2000 – 2002 |
| Dobesh PP et al[45]/2009 | USA | Registry of one medical center | NA | Retrospective cohort study. | 188 | 60 | 128 | January 2005**–**  December 2006. |
| Almog Y et al[46]/2004 | Israel. | Registry of one medical center | NA | Prospective observational cohort study | 361 | 82 | 279 | January 2003–  September 2003, |
| Mortensen EM et al[47]/2007 | USA | Veterans Affairs National Patient Care Database | YES | Retrospective national cohort study. | 3,018 | 480 | 2,538 | October 1998–  September 1999 |
| Park S W et al[48]/2013 | Korea | NA | YES | Retrospective cohort study | 949 | 199 | 750 | February 2005 and June 2012 |

Frost FJ et al[40] including a matched cohort study and a separate case–control studies have been counted as two studies; NA, not available
