# Supplementary material for: Effect of statin therapy on mortality from infection and sepsis: a meta-analysis of randomized and observational studies
Source: Crit Care. 2014 Apr 11;18(2):R71. doi: 10.1186/cc13828 (PMC4056771; doi:10.1186/cc13828)
Supplement: Additional file 2: Table S2 — Outcomes of observational studies reviewed. [file cc13828-S2.doc]

**Additional table 2. Outcomes of Observational Studies Reviewed**

| **Study/Year** | **Mean Age(y)**  **Statin/**  **Non-statin** | **Male Sex(%)**  **Statin/ Non-statin** | **APACHE II Score**  **Statin/**  **No-statin** | **Study Population** | **Statin Exposure** | **Main Outcomes** | **Conclusion** |
| --- | --- | --- | --- | --- | --- | --- | --- |
| Rothberg MB et al [22]/2012 | 72.4±11.5/  69.6± 16.7 | 49.2/42.7 | NA | Pneumonia | At least one dose of any statins on hospital day 1 or 2 | All-cause, and in-hospital  mortality. | Inpatient treatment with statins is associated with a modest reduction in pneumonia mortality outside of intensive care. |
| Yende S et al[23]/ 2011 | 71.6 ± 11.1/  66.0± 17.9 | 54.2/51.3 | NA | CAP and sepsis | Statin^*^ | Inflammation markers; ninety-day mortality | We found no evidence of a protective effect for statin use on clinical outcomes and only modest differences in circulating biomarkers in community-acquired pneumonia. |
| Forrest GN et al[24]/2010 | 65.9±9.4/  67.7± 8.7 | 73/40 | 14.9±3.8/  18.2 ±5.0 | Candidemia | Taking a statin (simvastatin,  atorvastatin or pravastatin) at the onset of candidemia, untill to the end of therapy | 30-day survival or discharge rate. | Statins may provide a survival benefit in candidemia. |
| Thomsen RW et al[25]/2008 | NA | 59.8/52.7 | NA | Pneumonia | Statin^*^ | All-cause death within 30 and 90 days | The use of statins is associated with decreased mortality after hospitalization with pneumonia |
| Donnino MW et al[26]/2009 | 73/56 | 48/51 | NA | Clinical infection | Statin^*^ | In-hospital mortality | Patients who were admitted to the hospital with infection and received statin therapy while hospitalized had a significantly lower in-hospital mortality compared to patients who did not receive a statin. |
| Doshi SM et al[27] 2013 | 68±10/ 63±12 | NA | NA | Pneumococcal Pneumonia | Statin^*^ | 7,14,20,30-day mortality | Patients who are receiving statins at the time of admission for pneumococcal pneumonia have better clinical outcomes than those who are not. Treatment with a macrolide does not appear to confer a survival benefit. |
| Yeh PS et al[28]/2012 | 73.3±10.6/ 74.3±11.5 | 47/49 | NA | Stroke-associated infection | Statin^*^ | 3 months mortality | Statin use was not associated with a better functional outcome or survival in patients with stroke-associated infection. |
| Goodin J et al[29]/2011 | 66.7±13.5/ 60.4±19.5 | 58.9/45.7 | NA | sepsis | Statin^*^ | hospital mortality; ICU mortality, hospital and ICU length of stay; mechanical ventilation and vasopressor therapy duration | This current retrospective study did not find any benefit of statin use on primary and secondary outcomes of the patients admitted to an academic hospital with sepsis. |
| Nseir W et al[30] /2012 | 68 ± 12/  64 ± 22 | 46/54 | NA | Bacteremic patients | Simvastatin 20–40 mg daily | 30-day in-hospital  mortality | Statins reduce the 30-day in-hospital all-cause mortality of bacteraemic patients. Long-term statin use prior to the bacteraemia improves the survival of bacteraemic patients more than short-term statin use. |
| Williams JM et al[31]/2011 | 72/46 | 56.5/ 50.4 | NA | Infection | Statin^*^ | 30 day in-hospital mortality | These data do not support an independent association between current preadmission statin use and lower 30-day in-hospital mortality in emergency patients admitted with infection. |
| Myles PR et al [32]/2009 | 74/56 | 53.9/46.1 | NA | Pneumonia | Statin^*^ | 30-day mortality; 2.8 years all-cause mortality | The use of statins is associated with a lower risk of short and long-term mortality following pneumonia. |
| Chalmers JD et al[33]/2008 | 74/56 | 53.9/46.1 | NA | CAP | Simvastatin; or  Atorvastatin or  pravastatin; | 30-day mortality; need for mechanical ventilation; the development of complicated pneumonia. | Statin use is associated with reduced markers of systemic inflammation and improved outcomes in patients admitted with community-acquired pneumonia. |
| Mortensen EM et al[34] /2012 | 74.8 ± 6.5  /74.8 ± 6.8 | 98.2/98.2 | NA | Pneumonia;  influenza; sepsis | atorvastatin; or cerivastatin, or  fluvastatin, or lovastatin, or pravastatin, or simvastatin. | 30-day all-cause mortality; use of invasive mechanical ventilation, length of hospital stay, and vasopressor use | Statins, and to a lesser extent ACE inhibitors and ARBs, are associated with improved pneumonia-related outcomes. |
| Leung S et al[35]/2012 | 69.5±13.4/  62.3±17.9 | 50.2/48.4 | 16/15 | Bloodstream infection | Simvastatin or atorvastatin or pravastatin or  Lovastatin or  rosuvstatin | 90-day all-cause mortality, use of mechanical ventilation and non-invasive positive pressure ventilation; hospital length of stay | After adjusting for the propensity to receive statin therapy, no statistically significant association between statin therapy prior to bloodstream infection and survival was identified. |
| Yang KC et al[36] /2007 | 65±14/  64±18 | 44.2/57.7 | 13.7±1.7/  15.9±0.9 | Sepsis | Simvastatin  (0-40mg/d);  atorvastatin (10-30mg/d); pravastatin (10-30mg/d); fluvastatin (20-40mg/d)  ; lovastatin (20-40mg/d) | Mortality at 30 days, 30-day; sepsis-related mortality | Short-term, sepsis-related mortality in a septic Taiwanese population was not reduced with statin treatment in our study. |
| Kruger P et al[37] 2006 | NA | NA | NA | Bacteraemic patients. | Simvastatin (36%);  atorvastatin  (22%); pravastatin (8%). | All-cause hospital mortality; death attributable to bacteraemia | This retrospective study demonstrates a significant survival benefit associated with continuing statin therapy in bacteraemic patients. |
| Hsu J et al[38]/2009 | NA | NA | NA | Bloodstream infections | Statin^*^ | Clinical cure and death rate within the 15-day | Appropriate antibiotic therapy and statin use are associated with lower risk of mortality from BSIs in this patient population. |
| Mortensen EM et al[39]/2008 | 74.6±5.9/  75.7 ±6.3 | 98/99 | NA | CAP | Statin^*^ | 30-day mortality | The present study finds that prior outpatient use of statins and, to a lesser extent, angiotensin-converting enzyme inhibitors, is associated with lower mortality for subjects hospitalised with community-acquired pneumonia |
| Frost FJ et al[40]/ 2007 | NA | NA | NA | Pneumonia；  Influenza; COPD | low daily dose (<4 mg/d) vs  moderate daily dose (≥4 mg/d). | Inpatient mortality | This study found a dramatically reduced risk of COPD death and a significantly reduced risks of influenza death among moderate-dose statin users. |
| Frost FJ et al [40]/2007 | NA | NA | NA | Influenza | low daily dose (<4 mg/d) vs  moderate daily dose (≥4 mg/d). | Inpatient mortality | This study found a dramatically reduced risk of COPD death and a significantly reduced risks of influenza death among moderate-dose statin users. |
| Liappis AP et al[41]/ 2001 | 63.7/ 63.2 | 100/99.4 | NA | Bacteremic infections | Simvastatin;  fluvastatin;  atorvastatin | Hospital mortality | These data suggest a potential clinical role of statins in bacteremic infection; however, the mechanism by which mortality is reduced remains undefined. |
| Mortensen EM et al[42] /2005 | 66.3±12.3/59.4±16.8 | 84/78 | NA | CAP | Statin^*^ | 30-day mortality | Prior outpatient statin use was associated with decreased mortality in patients hospitalized with community-acquired pneumonia despite their use being associated with comorbid illnesses likely to contribute to increased mortality. |
| Thomsen RW et al[43]/2006 | NA | 59/55 | NA | Bacteremia | Simvastatin(48%);  Pravastatin(28%);  Atorvastatin(18%);  other statins  [13%] | 30 and 180 days mortality rates | This study provides evidence against the hypothesis that statin use has an effect on short-term mortality after bacteremia. Statin use was, however, associated with a substantially decreased mortality between 31 and 180 days after bacteremia. |
| Majumdar SR et al[44]/2006 | NA | 56/53 | NA | CAP | Simvastatin;  pravastatin; atorvastatin; | Composite of in-hospital mortality or admission to an ICU. | Statins are not associated with reduced mortality or need for admission to an intensive care unit in patients with pneumonia. |
| Dobesh PP et al[45]/2009 | 68.7±12.6/ 65.4± 13.3 | 53.3/ 53.1 | 25.8±5.1/  26.0±5.9 | Severe sepsis | Statin^*^ | Inpatient mortality; ICU length of stay; total hospital cost . | The use of statins was associated with a protective effect. Patients with severe sepsis,as demonstrated by a significant reduction in mortality compared with patients not receiving statins. |
| Almog Y et al[46]/2004 | 70.4±8.8/  69.6±12.5 | 45.1/  49.5 | 10.8±8.1/  11.1±6.9 | bacterial infection | Simvastatin (69.5%)  Pravastatin (20.7%)  Other statin (9.8%) | The rate of severe sepsis and ICU admission, 28 day mortality rate | Prior therapy with statins may be associated with a reduced rate of severe sepsis and ICU admission. |
| Mortensen EM et al[47]/2007 | 73.1±5.5/  74.7 ± 6.2 | 98/99 | NA | Sepsis | Atorvastatin  cerivastatin, fluvastatin, lovastatin, pravastatin,  simvastatin. | 30-day mortality. | Use of statins and/or ARBs before admission was associated  with decreased mortality in patients hospitalized with sepsis. |
| Park SW et al[48]/2013 | 65.9±14.2/ 62.5± 16.1 | 46.2/55.2 | NA | Clostridium  difficile infection | Statin^*^ | Average symptom recovery time, 30-day mortality. | Prior statin exposure in patients with clostridium  difficile infection is associated with a successful response to treatment. |

Frost FJ et al[40] including a matched cohort study and a separate case-control studies have been counted as two studies; NA, not available; APACHE II, the acute physiology and chronic health evaluation II; Statin^*^, exact types of statins, daily dose are not described; CAP, community acquired pneumonia; COPD, chronic obstructive pulmonary disease; ICU, intensive care unit; ARB, angiotensin receptor blocker.
