# Supplementary material for: Effect of statin therapy on mortality from infection and sepsis: a meta-analysis of randomized and observational studies
Source: Crit Care. 2014 Apr 11;18(2):R71. doi: 10.1186/cc13828 (PMC4056771; doi:10.1186/cc13828)
Supplement: Additional file 3: Table S3 — Main outcomes of observational studies reviewed. [file cc13828-S3.doc]

**Additional table 3. Main Outcomes of Observational Studies Reviewed**

| **Author/ Year** | **OR /HR and 95% CI on multivariate analysis** | **Adjusted covariates** | **Undjusted data** | | | | **Type of mortality** | **Definition of stains** |
| --- | --- | --- | --- | --- | --- | --- | --- | --- |
|  |  |  | NO.  Statin (deaths) | NO.  Statin (survivals) | NO.  Non-statin (deaths) | NO.  Non-statin (survivals) |  |  |
| Rothberg MB et al [22]/2012 | (OR, 0.90; 95% CI, 0.82-0.99) | Demographics, comorbidities, severity of illness | 901 | 22,384 | 5,617 | 92,352 | In-hospital mortality | Current statin use (taking any kind of statins on hospital day 1 or 2) |
| Yende S et al[23]/ 2011 | (OR, 0.90; 95% CI, 0.63-1.29) | Age, comorbidity, and illness severity/propensity score | 39 | 387 | 176 | 1,293 | 90-day mortality | Former statin use (defined as a history of statin use in the week before admission) |
| Forrest GN et al[24]/2010 | (OR, 0.22; 95% CI, 0.22-2.4) | Demographics, comorbidities，severity of illness and statin therapy | 4 | 9 | 19 | 11 | 30-day mortality | Current statin use (patients take a statin (simvastatin, atorvastatin, or pravastatin) at the onset of candidemia, untill to the end of therapy) |
| Thomsen RW et al[25]/2008 | (OR, 0.69; 95% CI, 0.58-0.82) | Age, sex, comorbidity, alcoholism, use of immunosuppressive drugs, and use of preadmission, antibiotic agents, calendar period, socioeconomic markers, and other pro-phylactic cardiovascular drugs ,propensity score | 141 | 1,231 | 4,489 | 28,528 | 30-day mortality | Former statin use ( patients who filled at least 1 statin prescription more than 125 days before the hospitalization ) |
| Donnino MW et al[26]/2009 | (OR, 0.27; 95% CI, 0.1-0.72) | Gender, severity of illness, charlson comorbidity index, and duration of statin therapy | 9 | 465 | 70 | 1,492 | In-hospital mortality | Current statin use (patients receive statin therapy during their inpatient hospital course) |
| Doshi SM et al[27] 2013 | (HR, 0.39; 95% CI, 0.16-0.92) | Age, race, comorbidities, bacteremic status, alcohol use, length of stay and disease severity determined | 7 | 83 | 38 | 219 | 30-day mortality | Current statin use (patients receive statins at the time of admission) |
| Yeh PS et al[28]/2012 | (HR, 1.68; 95% CI, 0.79-3.56) | Age, sex, statin use, atrial fibrillation, creatinine, cholesterol, severity of illness, and charlson index, Propensity score | 16 | 105 | 62 | 331 | 90-day mortality | Current statin use (patients had not received statin treatment prior to admission |
| Goodin J et al[29]/2011 | (OR, 1.36; 95% CI, 0.59-3.13) | Age, Gender, body mass index，ischaemic heart disease, Smoking history, sequential organ failure assessment score | 21 | 103 | 87 | 357 | in-hospital mortality | Current statin use (statin at time of admission to hospital) |
| Nseir W et al[30] /2012 | (HR, 0.34; 95% CI, 0.18-0.62) | Age, Diabetes mellitus, hypertension,chronic renal failure, ischemic heart disease, chronic obstructive pulmonary disease, charlson comorbidity index | 20 | 138 | 39 | 122 | 30-day mortality | Former statin use (prior statin use) |
| Williams JM et al[31]/2011 | (OR, 0.96; 95% CI, 0.55-1.69) | Age, severity of disease, comorbid status, and propensity score | 24 | 371 | 65 | 2,182 | 30-day mortality | Former statin use (prior statin use) |
| Myles PR et al [32]/2009 | (HR, 0.33; 95% CI, 0.19-0.58) | Age, sex, townsend’s deprivation score, current smoking and charlson comorbidity index score and coprescription of other exposure drugs | 45 | 312 | 860 | 2,464 | 30-day mortality | Current statin use (the most recent prescription was within 30 days before the pneumonia index date.) |
| Chalmers JD et al[33]/2008 | (OR, 0.46; 95% CI, 0.25-0.85) | Age, pneumonia severity , comorbidity [chronic cardiac failure, cerebrovascular disease, chronic renal failure, chronic obstructive pulmonary disease, diabetes mellitus], and smoking status. | NA | NA | NA | NA | 30-day mortality | Former statin use (prior statin use) |
| Mortensen EM et al[34] /2012 | (OR, 0.74; 95% CI, 0.68-0.82) | Sociodemographic variables, and receipt of guideline concordant antibiotics, comorbid conditions, and other medications. | NA | NA | NA | NA | 30- day mortality | Former statin use (prior statin use) |
| Leung S et al[35]/2012 | (HR, 0.86; 95% CI, 0.70-1.06) | Age, gender, race, body mass index and charlson’s score | 89 | 503 | 337 | 1,210 | 90-day mortality | Current statin use ( administration of any statin medication at the time blood culture was sampling and/or documentation of statin use as an outpatient prior to hospitalization if the bacteremic blood culture was drawn within 24 hours of admission) |
| Yang KC et al[36] /2007 | NA | NA | 20 | 84 | 66 | 284 | 30-day mortality | Former statin use (patients take a statin at least 30 days before the sepsis explosion and continuing to receive statin therapy during the hospital course ) |
| Kruger P et al[37] 2006 | (OR, 0.39; 95% CI, 0.17-0.91) | NA | 7 | 59 | 86 | 286 | In-hospital mortality | Former statin use ( prior outpatient use of statins) |
| Hsu J et al[38]/2009 | (OR, 0.18; 95% CI, 0.04-0.78) | Age, gender, severity of illness, comorbidities [dialysis, liver disease], statin use,clinical features [hypothermia, fever, hypotension , mental status changes, Intensive care unit care, pressors], multidrug-resistant pathogen, appropriate antibiotic therapy, and origin of bacteremia | 4 | 74 | 40 | 193 | 15-day mortality | Current statin use ( taking a statin at the time of BSI (intra-abdominal solid-organ transplants, bloodstream infections ) explosion |
| Mortensen EM et al[39]/2008 | (OR, 0.54; 95% CI, 0.42-0.70) | Appropriate propensity score and hospital admission | 78 | 1,489 | 772 | 6,313 | 30-day mortality | Former statin use (prior statin use) |
| Frost FJ et al[40]/ 2007 | (HR, 0.61; 95% CI, 0.41-0.92) | Sex, birth year, and duration of phase 2 enrollment in all models. | NA | NA | NA | NA | In-hospital mortality | Former statin use (at least 90 days of cumulative statin exposure prior to death) |
| Frost FJ et al [40]/2007 | (OR, 0.62; 95% CI, 0.43-0.91) | Sex, birth year, and duration of phase 2 enrollment in all models. | NA | NA | NA | NA | In-hospital mortality | Current statin use ( the daily dose (4 mg/d) for a 3-month to 1-year period following initiation of statin therapy, |
| Liappis AP et al[41]/ 2001 | (OR, 7.6; 95% CI, 1.01-57.5) | Age, comorbid conditions, concurrent medications, site of infection, vital signs, ICU stay, and laboratory data | 2 | 33 | 100 | 253 | In-hospital mortality | Current statin use (patients take a statin at the time of admission and continue statin throughout the course of hospitalization) |
| Mortensen EM et al[42] /2005 | (OR, 0.36; 95% CI, 0.14-0.92) | Propensity score, use of statin at presentation, and process of care measures | NA | NA | NA | NA | 30-day mortality. | Former statin use ( prior outpatient use of statins) |
| Thomsen RW et al[43]/2006 | (OR, 0.93; 95% CI, 0.66-1.3) | Gender, age group, level of comorbidity, alcohol-related conditions, use of immuno-suppressive drugs and systemic antibiotics | 35 | 141 | 118 | 5,059 | 30-day mortality. | Former statin use (persons filling at least one prescription for statins within 1 year before the date of hospitalization with bacteremia.) |
| Majumdar SR et al[44]/2006 | (OR, 1.1; 95% CI, 0.76-1.60) | Age, sex, nursing home resident, selected comorbidities, number of drugs, smoking status, independent mobility, immunisations, data specific to community acquired pneumonia, propensity score | 25 | 300 | 309 | 2,781 | In-hospital mortality | Former statin use (use of statins for at least one week before admission and during hospital stay) |
| Dobesh PP et al[45]/2009 | (OR, 0.42; 95% CI, 0.21-0.84) | Statin use, age, sex, and severity of illness | 19 | 41 | 62 | 66 | In-hospital mortality | Current statin use ( receiving any statin at the time of admission) |
| Almog Y et al[46]/2004 | (OR, 0.07; 95% CI, 0.01-0.51) | Preexisting conditions [hypertension, chronic ischemic heart disease, chronic heart failure , diabetes, chronic renal failure] and lipids profile. | 3 | 79 | 24 | 255 | 28-day mortality. | Former statin use (taking statins for 1 month before their admission) |
| Mortensen EM et al[47]/2007 | (OR, 0.48; 95% CI, 0.36-0.64) | Charlson comorbidity index score, age, sex, marital status, race-ethnicity, and counts of drugs from each class of potentially confounding drugs | 79 | 401 | 732 | 1,806 | 30-day mortality | Former statin use (receiving at least one active and filled prescription within 90 days of admission) |
| Park SW et al[48]/2013 | NA | NA | 10 | 189 | 31 | 719 | 30-day mortality | Former statin use (statin users who initiated therapy >12 weeks before the index date) |
| Frost FJ et al[40] including a matched cohort study and a separate case-control studies have been counted as two studies; NA:Not Applicable ;OR, Odds Ratio; HR, Hazard Ratio; 95%CI, 95% Confidence Intervals; NA, Not Available . | | | | | | | | |
